# Primary T-Cell Non-Hodgkin Lymphoma of the Vagina

**DOI:** 10.1155/2015/893083

**Published:** 2015-05-26

**Authors:** J. L. Herraiz, A. Llueca, Y. Maazouzi, D. Piquer, A. Palmeiro, E. Calpe

**Affiliations:** ^1^Department of Obstetrics and Gynecology, General University Hospital of Castellon, Avenida Benicassim S/N, Castellón de la Plana, 12004 Castelló, Spain; ^2^Department of Pathology, General University Hospital of Castellon, Avenida Benicassim S/N, Castellón de la Plana, 12004 Castelló, Spain

## Abstract

The primary vaginal T-cell non-Hodgkin lymphoma is a rare form of lymphoma. Most of the previously published cases were about B-cell non-Hodgkin lymphomas. We present the case of a vaginal mass in an 82-year-old patient presenting vaginal bleeding. The results of the immunohistological studies of the mass revealed the presence of a cytotoxic T-cell non-Hodgkin lymphoma, which is the least common subtype.

## 1. Introduction

The primary affectation of the female genital tract in non-Hodgkin lymphomas is rare and represents 1.5–2% of all extraganglionar primary lymphomas. The most commonly affected areas are in the ovaries (50%) and cervix, while the uterine and vaginal affectation remains extremely rare (6%) [1].

The average age of onset is in the fifth decade of life [2, 3] and the most common histology is usually that of diffuse large B-cell lymphoma (DLBCL). The patient's motive for consultation is usually due to abnormal vaginal bleeding [4].

Despite the lack of standard treatments for the non-Hodgkin vaginal lymphoma, various treatment algorithms are used which include chemotherapy and radiotherapy, eliciting good results in initial stages of the disease [5].

We hereby present an unusual case of a vaginal T-cell lymphoma. Most of the reported cases were of B-cell non-Hodgkin lymphomas [1, 6].

## 2. Case Presentation

**Figure 1:**
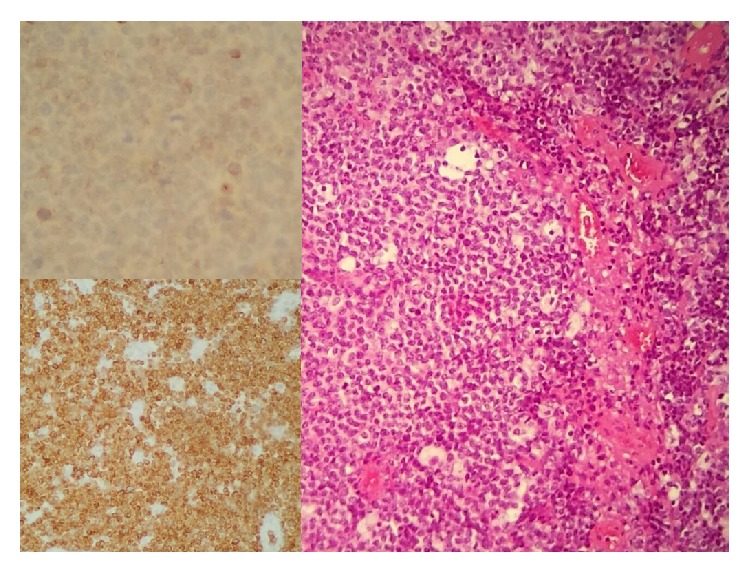
Tissue fragment where medium-sized cells and granular nucleus are observed, along with vascular destruction. The images on the left show Cd56 (−) and EBV (+) staining.

**Figure 2:**
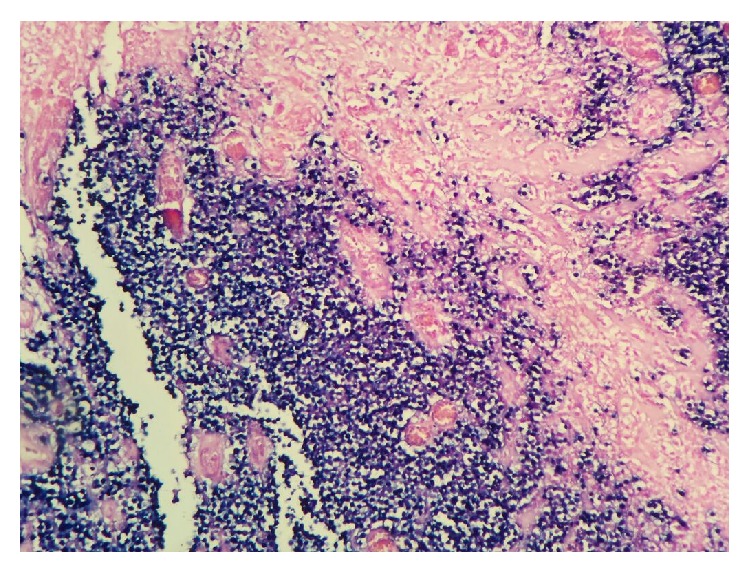
Visualization of positive Epstein-Barr staining.

We present the case of an 82-year-old patient with no personal history of interest that came for a consultation in our gynecology department prompted by an episode of vaginal bleeding lasting various days. Her obstetrical/gynecological background includes 2 vaginal births and menopause at the age of 50. During the exploration, we found a mass, hard and friable in consistency, occupying the entire vaginal cavity. We biopsied the tumor, and the histologic results revealed the presence of medium-sized cells with granular nuclei and vascular destruction accompanied by necrosis. The immunohistochemistry showed CD3 (+), CD56 (−), CD8 (−), EBV (+), CD4(+), and granzyme B (+). The studies' conclusions were that of a NK/cytotoxic T-cell extranodal lymphoma (Figures 1 and 2).

**Figure 3:**
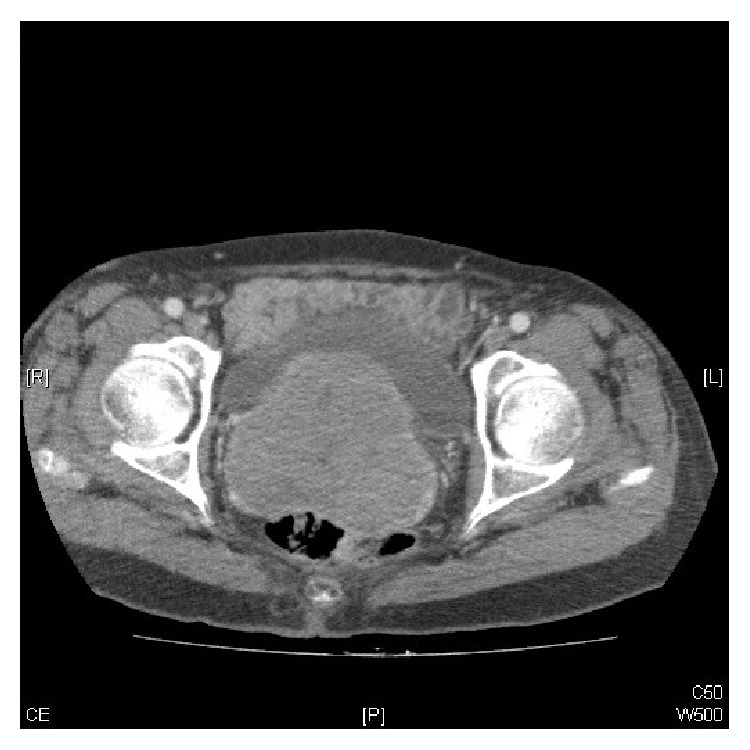
CT scan demonstrating a large mass occupying the vaginal cavity.

**Figure 4:**
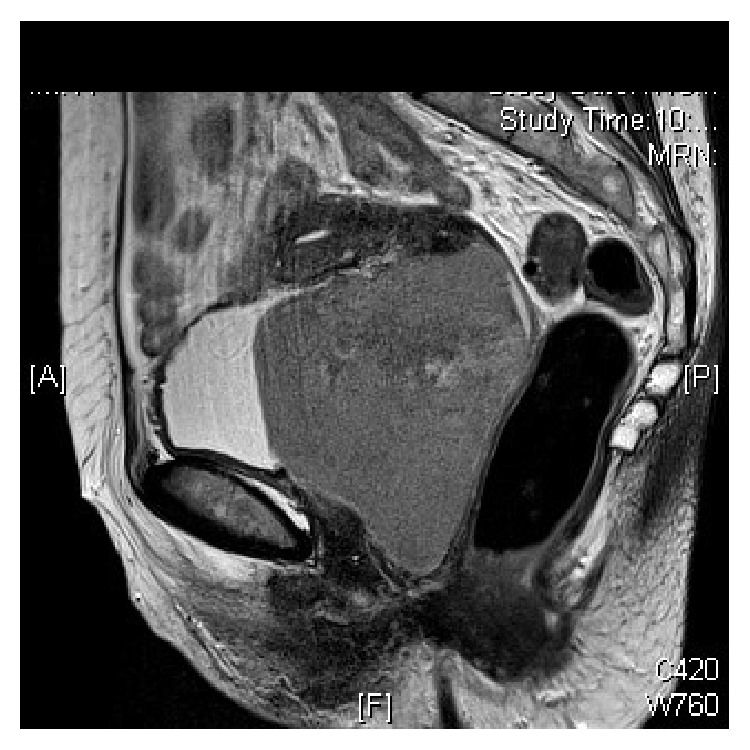
MRI demonstrating the vaginal lesion displacing the uterus towards the cranial direction.

**Figure 5:**
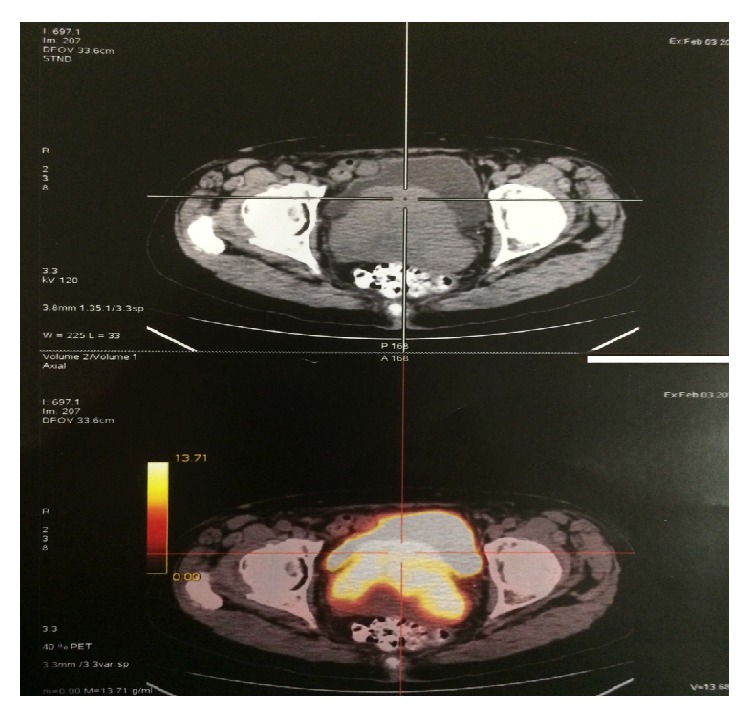
PET scan showing the mass in the vesical-uterine space.

A thoracoabdominal pelvic CT scan was performed, confirming the solid mass described in our exploration: a 7.5 × 7.5 × 9 cm mass with a rounded morphology, occupying the vesical-uterine space. There were no pathological findings in the uterus and ovaries. There was no sign of retroperitoneal or pelvic lymphadenopathies, nor were there lesions at the extrapelvic level (Figure 3). The Magnetic Resonance Imaging (MRI) corroborated the findings at the vesical-uterine level, which were previously mentioned in the CT scan results (Figure 4). The PET scan showed the previously discovered mass in the vesical-uterine space, presenting a high metabolic activity (Figure 5). The rest of the studies showed no evidence of increased tumor extension. The complete blood count (CBC), LDH, B2 microglobulin, and albumin levels were within the range of normality.

The patient rejected any type of treatment. She received palliative care at home and died two months after having been diagnosed.

## 3. Discussion

The Lymphomas are divided into two main categories: Hodgkin lymphoma (HL) and non-Hodgkin lymphoma (NHL). The latter is mainly currently divided into Precursor Lymphoid Neoplasms, B-cell neoplasms, and mature T/NK-Cell lymphomas. The incidence of non-Hodgkin lymphoma is 2–5% of all cancers [7].

Primary NHL manifestation in the female genital tract is extremely rare, with a prevalence ranging between 0.2 and 1.1%. Secondary affectation of the genital tract due to the dissemination of a lymphoma is around 7–30% of all cases. The most frequent location in extranodal NHL cases with genital affectation is the ovaries, followed by the cervix and uterus, and, lastly, the most unusual location is the vagina [8–10]. The most common histological subtype of gynecological lymphomas, primary or secondary, is the Large B-cell diffuse lymphoma [11]. The cytotoxic T-cell NHL, such as the one presented in our case, is extremely rare.

The incidence of extranodal lymphomas has increased in the last decades [4]. This increase may be justified by the association of different infectious agents, such as the Epstein-Barr virus [12], the human immunodeficiency virus [13, 14], immunosuppressive therapies [15], and exposure to toxic substances and pollutants. Additionally, this increase may be also related to the new diagnostic techniques which keep on progressing [6, 12, 16].

The mean age at onset for NHL is 50 years, with most of the women having entered menopause.

The most common form of presentation of this neoplasm is the abnormal vaginal bleeding; nevertheless, it may manifest itself as abdominal or perineal pain, dyspareunia, dysmenorrhea, and urethral obstruction [6]. It usually appears as a large, fast growing and endophytic mass. In our case, the patient consulted due to vaginal bleeding as the sole symptom and it presented itself as an exophytic mass.

The diagnosis is confirmed by biopsy. The material obtained through biopsy must be sufficient in order to perform conventional histology and immunohistochemical and molecular genetics studies to determine the lymphoma subtype [6, 17, 18].

The most important prognostic factor for survival is the tumor staging. The Modified Ann Arbor classification is used for the extranodal NHL staging [19] (Table 1). According to this classification, the case we have presented corresponds to a stage IE. The majority of these tumors (75%) present themselves in stages IE.

After the Ann Arbor classification, a prognostic index for better survival was created for the extranodal NHL, which was more widely accepted [20]. Namely, the International Prognostic Index (IPI), which takes into account the age of the patient, the stage (Ann Arbor classification), number of extranodal sites affectation, the general condition of the patient, and the LDH levels.

The prognosis for the vaginal lymphoma is encouraging, with an mean survival of 5 years depending on the tumor stages: 80% during the initial stages (I and II) and 30% in advanced stages (III and IV) [1, 21].

The treatment of this type of tumor is not clearly established because of its low incidence. Radiotherapy has been used as the sole treatment in some cases, while chemotherapy has been used in others [22]; nevertheless, it seems that the combination of both treatments is the most accepted alternative [5]. Surgery may also be used in treatment-resistant cases associated with chemotherapy, although its role is not generally accepted.

In our case, none of these treatment options were used due to the patient's refusal.

## 4. Conclusion

The incidence of the vaginal lymphoma is very low; however, it can demonstrate early symptoms suggesting a gynecologic neoplasm. Both gynecologist and pathologist must include this disease in the differential diagnosis of a vaginal mass so as to not delay or establish the wrong diagnosis that could lead to failure to treat these patients.

## Figures and Tables

**Table 1:** Staging system for extranodal lymphomas. Modified Ann Arbor classification.

Stage	Description
IE	Involvement of a single lymph node region or a single organ.

IIE	Involvement of a single extralymphatic organ or site and one or more regional lymph nodes ipsilateral to diaphragm.

IIE	Involvement of a single extralymphatic organ or site and regional lymph nodes on both sides of the diaphragm.

IVE	Diffuse involvement or dissemination of one or more extralymphatic sites, with or without the involvement of lymphatic nodes.
